# Design and validation of a multi-objective waypoint planning algorithm for UAV spraying in orchards based on improved ant colony algorithm

**DOI:** 10.3389/fpls.2023.1101828

**Published:** 2023-02-02

**Authors:** Haoxin Tian, Zhenjie Mo, Chenyang Ma, Junqi Xiao, Ruichang Jia, Yubin Lan, Yali Zhang

**Affiliations:** ^1^ College of Engineering, South China Agricultural University, Guangzhou, China; ^2^ Guangdong Laboratory for Lingnan Modern Agriculture, Guangzhou, China; ^3^ National Center for International Collaboration Research on Precision Agricultural Aviation Pesticide Spraying Technology, Guangzhou, China; ^4^ College of Aerospace Engineering, Nanjing University of Aeronautics and Astronautics, Nanjing, China; ^5^ College of Electronic Engineering, College of Artificial Intelligence, South China Agricultural University, Guangzhou, China

**Keywords:** UAV, waypoint planning, multi-objective, ant colony optimization, orchard plant protection

## Abstract

**Introduction:**

Current aerial plant protection with Unmanned Aerial Vehicles (UAV) usually applies full coverage route planning, which is challenging for plant protection operations in the orchards in South China. Because the fruit planting has the characteristics of dispersal and irregularity, full-coverage route spraying causes re-application as well as missed application, resulting in environmental pollution. Therefore, it is of great significance to plan an efficient, low-consumption and accurate plant protection route considering the flight characteristics of UAVs and orchard planting characteristics.

**Methods:**

This study proposes a plant protection route planning algorithm to solve the waypoint planning problem of UAV multi-objective tasks in orchard scenes. By improving the heuristic function in Ant Colony Optimization (ACO), the algorithm combines corner cost and distance cost for multi-objective node optimization. At the same time, a sorting optimization mechanism was introduced to speed up the iteration speed of the algorithm and avoid the influence of inferior paths on the optimal results. Finally, Multi-source Ant Colony Optimization (MS-ACO) was proposed after cleaning the nodes of the solution path.

**Results:**

The simulation results of the three test fields show that compared with ACO, the path length optimization rate of MS-ACO are 3.89%, 4.6% and 2.86%, respectively, the optimization rate of total path angles are 21.94%, 45.06% and 55.94%, respectively, and the optimization rate of node numbers are 61.05%, 74.84% and 75.47%, respectively. MS-ACO can effectively reduce the corner cost and the number of nodes. The results of field experiments show that for each test field, MS-ACO has a significant optimization effect compared with ACO, with an optimization rate of energy consumption per meter of more than 30%, the optimization rate of flight time are 46.67%, 56% and 59.01%, respectively, and the optimization rate of corner angle are 50.76%, 61.78% and 71.1%, respectively.

**Discussion:**

The feasibility and effectiveness of the algorithm were further verified. The algorithm proposed in this study can optimize the spraying path according to the position of each fruit tree and the flight characteristics of UAV, effectively reduce the energy consumption of UAV flight, improve the operating efficiency, and provide technical reference for the waypoint planning of plant protection UAV in the orchard scene.

## 1 Introduction

As of 2021, China’s total fruit output was 299.702 million tons, of which citrus output is 55.9561 million tons, ranking first in China ‘s fruit output (Stasistics, 2022). In the process of citrus production, pests and diseases are the main factors affecting the yield and safety of citrus (Du et al., 2011; Bassanezi et al., 2020), and efficient pest control methods are an indispensable and important link to ensure the yield and quality of citrus fruits (Yi, 2007; Zhang et al., 2014; Garcia et al., 2022). In recent years, with the development of precision agriculture, UAVs for plant protection have attracted widespread attention (Lan et al., 2019; Ru et al., 2020). The UAVs can take off and land vertically, with simple operation, high maneuverability and no terrain restrictions (Zhou et al., 2013; Yang et al., 2018), providing a new direction for the development of aerial plant protection technology.

In the research of UAV for plant protection, route planning is one of the key technologies to be solved urgently (Lan et al., 2017; Zhou et al., 2017). Local optimization for operating areas and routes is also a research hotspot for mobile robots (Zhao et al., 2017; Zhang et al., 2018). Liu et al. (2020) developed a short-range, accurate coverage route planning algorithm for aerial spraying with full coverage. In this method, the optimal route is obtained by analyzing the spraying voyage outside the spraying operation area and combining with the full-coverage route planning in order to avoid the problems of repeated and missed application in aerial pesticide application. Han et al. (2021) proposed an infield path planner that can automatically generate pattern-based X-turn path diagrams to provide efficient operation routes for polygonal rice fields. Xu et al. (2020) proposed a UAV route design algorithm for farmland with obstacles. The algorithm uses the characteristic information of the waypoints and combines the hybrid particle swarm algorithm to sort the waypoints, and designs a full-coverage route plan that can effectively avoid obstacles. These studies have carried out local optimization of the route according to the geometry of different farmlands, effectively improving the route coverage and ensuring the operation efficiency. However, this type of research is mainly aimed at densely planted rice field crops, and the operation method is mainly full-coverage reciprocating pesticide application. In the citrus orchard environment, fruit trees are different from field and facility horticultural crops, and have the characteristics of larger plant size, large planting spacing, and non-row planting (Wu et al., 2021). The application of UAVs for full-coverage route spraying may cause repeated and missed applications, and increase operating costs. At present, there are few studies on the precision route planning of UAV in orchard environment. Therefore, it is very important to carry out research on route planning for precision pesticide application in citrus fruit trees.

Route planning is a combinatorial optimization problem. Based on the known environment and operation information, a reasonable movement route was planned according to multiple constraints (Zhang et al., 2020; Zhou and He, 2021). In the route planning research of the orchard environment, we should not only consider the influence of the operating area, but also integrate the flight characteristics of the UAV to plan an operation route suitable for UAV plant protection in orchard. Although rotary-wing UAVs are better able to perform aerial transitions regardless of the terrain, there are still problems that cannot be ignored (Zhu et al., 2022). For example, the turning and starting and stopping of UAV during flight will cause a large energy loss (Fan et al., 2019). Alternatively, the load and battery of the UAV will also affect the battery life (Abeywickrama et al., 2018; Lin et al., 2020). Researchers proposed different solutions to the limitations of rotary-wing UAVs. Li et al. (2019) reduced the flight energy consumption of UAVs during non-working periods and improved operational efficiency by integrating constraints such as the spraying amount of each sortie and the returning point. Peng et al. (2019) used the autonomous constant-speed flight of UAVs and the minimum turning radius constraints to design a route planning method for UAVs in irregular farmland to achieve full-area coverage. Constraints such as distance, energy consumption, and operating time are the optimal route methods often used by researchers, because these constraints directly affect the operating efficiency and cost of UAVs.

At present, commonly used route planning algorithms include artificial potential field method (Zhang et al., 2017), ant colony algorithm (Patle et al., 2019), A* algorithm (Liu et al., 2019), genetic algorithm (Zhao et al., 2021) etc. The artificial potential field method and the A* algorithm have the advantages of simple and small amount of calculation (Huo et al., 2018), and can be better combined with other algorithms to solve various optimization problems. However, the artificial potential field method is mainly used for local trajectory search, usually as an auxiliary algorithm for global path search (Sun et al., 2017; Lin et al., 2019). The A* algorithm can quickly solve the shortest path between the starting point and the ending point, but it is not suitable for independently solving the path optimization problem that traverses all nodes (Erke et al., 2020; Tang et al., 2021a). Genetic algorithm and ant colony algorithm are both bionic algorithms (Tang et al., 2021b). Among them, genetic algorithm has good global optimization ability, but its operation speed is slow and its search efficiency is low. The path search in the face of a large number of nodes is easy to fall into the local optimal solution, and the effect of solving the optimal path problem of traversing nodes is poor. ACO is a probabilistic algorithm that finds the optimal path through steps such as heuristic search, pheromone feedback, and distributed computing (Cao et al., 2021). This algorithm has good problem optimization ability, and is easy to combine with other algorithms, and is widely used to solve optimization problems such as traveling salesman and secondary allocation of resources (Ebadinezhad, 2020; Zhang et al., 2021b). For example, Liu et al. (2022) realized the plant protection route planning for many tea fields in hilly areas by combining genetic algorithm and ant colony algorithm. Cao et al. (2021) added the influence of agricultural machinery operation execution ability to the pheromone update mechanism of the ant colony algorithm, and realized the collaborative management of agricultural machinery. Therefore, this paper solves the route planning problem of plant protection drones in orchards by improving the ant colony algorithm.

In this study, aiming at the problems that UAV full coverage reciprocating routes cannot accurately cover every fruit tree, repeated application, missed application and high energy consumption, precision planning of UAV plant protection routes based on multi-objective tasks was carried out. By improving the ant colony algorithm and integrating the three constraints of distance factor, rotation angle factor and the number of task nodes, MS-ACO was proposed to improve the efficiency of UAV operation and reduce flight energy consumption. First, the corner factor is introduced into the heuristic function of ACO. At the same time, a ranking optimization mechanism is added to the pheromone update. Finally, node cleaning is performed on the optimized path to obtain the optimal path. The algorithm can effectively optimize the path corner and the number of nodes under the condition of ensuring the path length, and improve the efficiency of plant protection. The results provide a theoretical basis for the research on aerial precision plant protection technology in orchards.

## 2 Materials and methods

This study mainly includes four parts: environment modeling, algorithm improvement, simulation experiment, and field experiment. The first part is the overview of the test area and the establishment of the environmental model; the second part is the improvement of the algorithm, including heuristic information fusion, pheromone update strategy based on sorting and optimization of optimal path nodes; comparing the paths optimized by MS-ACO and ACO with the number of nodes to verify the feasibility of the improved algorithm; finally, we use the built UAV platform to conduct field experiments, and verify the real validity of the algorithm through the optimization of flight time, range, turning angle and energy consumption per meter degree.

### 2.1 Experimental area overview

**Figure 1:**
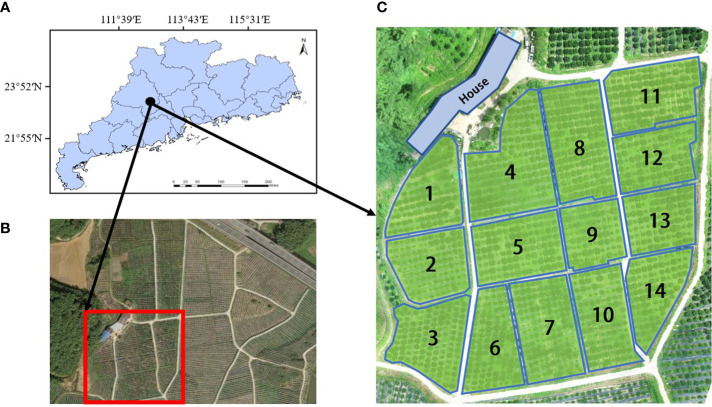
Experimental site and site division. **(A)** Geographic location of the experimental site, **(B)** Satellite map of the experimental area, **(C)** Division of the experimental area.

The experimental site is located in a citrus orchard in Sihui City, Guangdong Province, China (23°36′N, 112°68′E), as described in **Figure 1A, B**. The site is a near-plain terrain with an average slope of less than 5°, subtropical monsoon climate, and an average annual rainfall of about 1900 mm (Niu et al., 2021), which is suitable for citrus growth. The citrus trees in this orchard are mostly irregular terrain, divided by an irregular grid of 30×30m. The height of the fruit trees is between 2m and 5m, the average diameter of the crown is 3m, and the spacing between trees and rows is about 3m. We divided the experimental area into 14 plots according to the actual road distribution in the orchard, and selected the numbered 1, 4 and 6 for simulation experiments and field experiments, as shown in **Figure 1C**.

### 2.2 Environment modeling

**Figure 2:**
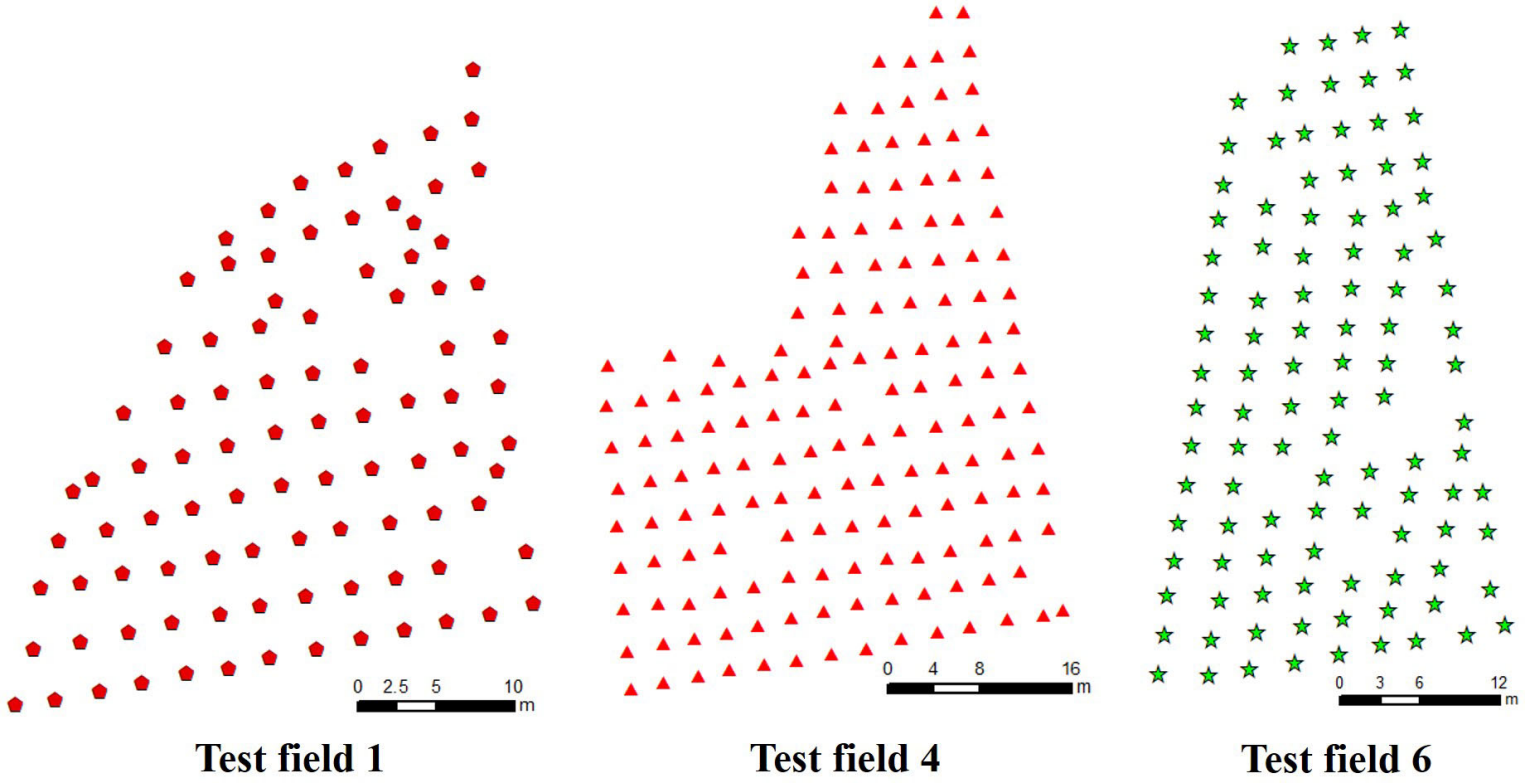
Extraction of citrus tree coordinates.

In order to ensure that the path optimized by the improved algorithm has practical application value, the coordinate system of our simulation environment was constructed by using the real geographic coordinate system of the orchard, and the simulation environment was constructed by extracting the geographic coordinate information of citrus trees. Since the experimental site of this study is a near-plain terrain with little difference in the height of the fruit trees, the effect of the difference between orchard elevation and tree height is not taken into account, and the coordinate system constructed is a two-dimensional plane coordinate system. At present, there are many methods for coordinate point extraction, such as manual marking, feature point extraction, and image fusion based on geographic information systems (Helmholz et al., 2014; Yu et al., 2020). The method based on manual extraction of coordinate points of fruit trees is cumbersome and not suitable for batch extraction of coordinates of citrus trees. The development of remote sensing images in recent years has make it possible to identify targets or large-scale target research in inaccessible environments (Hu et al., 2021; Ghaderizadeh et al., 2022; Jalayer et al., 2022; Zamani et al., 2022). For example, using satellite remote sensing images to detect the impact of climate change on planting patterns (Tariq et al., 2022a), drawing and detecting the distribution map of farmland and crop types (Tariq et al., 2022b), and large-scale extraction of farmland (Sharifi et al., 2022). Although this approach works very well with high spatial resolution. However, this method is suitable for obtaining large-scale farmland data, and is not suitable for extracting geographic coordinate information of a single fruit tree. Tian et al. (2022) proposed a method for extracting citrus tree coordinates based on YOLOv5s. First, an orchard orthophoto map is drawn using a remote sensing dataset. Secondly, the citrus tree image containing geographic information is identified by the YOLOv5s model and the pixel coordinates of the center of the citrus tree in the image are extracted. Finally, the geographic coordinates of citrus trees are extracted through spatial coordinate system transformation and Gauss Kruger inverse algorithm. This method can automatically identify and extract the geographic coordinates of citrus trees, and the accuracy of the extracted geographic coordinates can reach ±0.15m. It has the characteristics of high recognition and extraction accuracy, and simple and quick extraction steps. Therefore, this method was applied to extract the coordinate information of citrus trees. According to the planting situation of citrus trees and the number of plots, the coordinates of citrus trees were extracted from the three experimental fields numbered 1, 4, and 6 in **Figure 1C**, and the extracted coordinate information is shown in **Figure 2**. The coordinate points in the figure are the coordinates of each citrus tree, which is a set of world coordinates containing real geographic information, providing data support for subsequent simulation experiments and field experiments.

### 2.3 Ant colony optimization improvement

The path planned by the traditional ant colony algorithm has the advantage of the shortest path. However, for the complex orchard environment and the maneuvering characteristics of the UAV, the path optimized by the traditional ant colony algorithm has a large cumulative turning angle and a large number of redundant waypoints. This path is very unfriendly to the flight of the UAV, which will seriously affect the flight time of the UAV, increase the flight energy consumption, and reduce the operation efficiency of the UAV. In order to solve such problems, this study improved the traditional ant colony algorithm, including improving heuristic information, improving pheromone update mechanism and node optimization strategy.

#### 2.3.1 Ant colony optimization

The ACO was originally proposed to solve the traveling salesman problem, which is similar to the research content in this study, both of which are to solve the static task assignment problem. Taking the model as an example, there are n citrus trees in the UAV operation plot, that is, *n* tasks. Each ant needs to search from the first task, and select the next task according to the pheromone concentration on the path until all tasks are completed. The model records the path cost traversed by each ant after each iteration, and outputs the shortest path after all iterations are completed.

In this model, *m* is the number of ants, *d_ij_* (*i, j* = 1, 2,···,*n* represents the distance between task *i* and task *j*, and *τ_ij_* (*t*) represents the pheromone concentration between task *i* and task *j* at time *t*. At the initial moment, the pheromone concentration on each path is the same, that is, *τ_ij_* = 0 = *C* (*C* is a constant), and $P_{ij}^{k}\;$ represents the probability of the *K*th ant transferring from task *i* to task *j*, and the equation is as follows:


(1) $$P_{ij}^{k}=\{\;\;\;\begin{array}{c} \frac{\tau_{ij}^{\alpha }\left(t\right)\eta_{ij}^{\beta }\left(t\right)}{\sum_{s\epsilon allowed_{k}}\tau_{is}^{\alpha }\left(t\right)\eta_{is}^{\beta }\left(t\right)}\;\;\;\;\left(j\in allowed_{k}\right)\\ 0\;\;\;\;\;\;\;\;\;\;\;\;\;\;\;\;\;\;otherwise \end{array}$$

where $\eta_{ij}\left(t\right)=\frac{1}{d_{ij}}$ ; *allowed_k_* represents the set of tasks that ant k is allowed to access.

After each iteration, the pheromone concentrations on all paths will be updated again, and the pheromone update equation are as follows:


(2) $$\tau_{ij}\left(t+1\right)=\rho \tau_{ij}\left(t\right)+\Delta \tau_{ij}\left(t,t+1\right)$$

(3) $$\Delta \tau_{ij}\left(t,t+1\right)=\sum_{k=1}^{m}\Delta \tau_{ij}^{k}\left(t,t+1\right)$$

(4) Δτijk(t){QLk  Path (i,j) traversed by ant k  0     otherwise          

where $\Delta \tau_{ij}^{k}$ represents the pheromone concentration of the *K*th ant between task *i* and task *j* in this iteration; Δ*τ_ij_* (*t, t* + 1) represents the sum of the pheromone concentrations of all ants between task *i* and task *j* in this iteration; *Q* is the pheromone intensity; *ρ* is the pheromone volatile factor; *L_k_* is the total length of the path searched by ant *k* in this iteration.

#### 2.3.2 Heuristic information improvement

**Figure 3:**
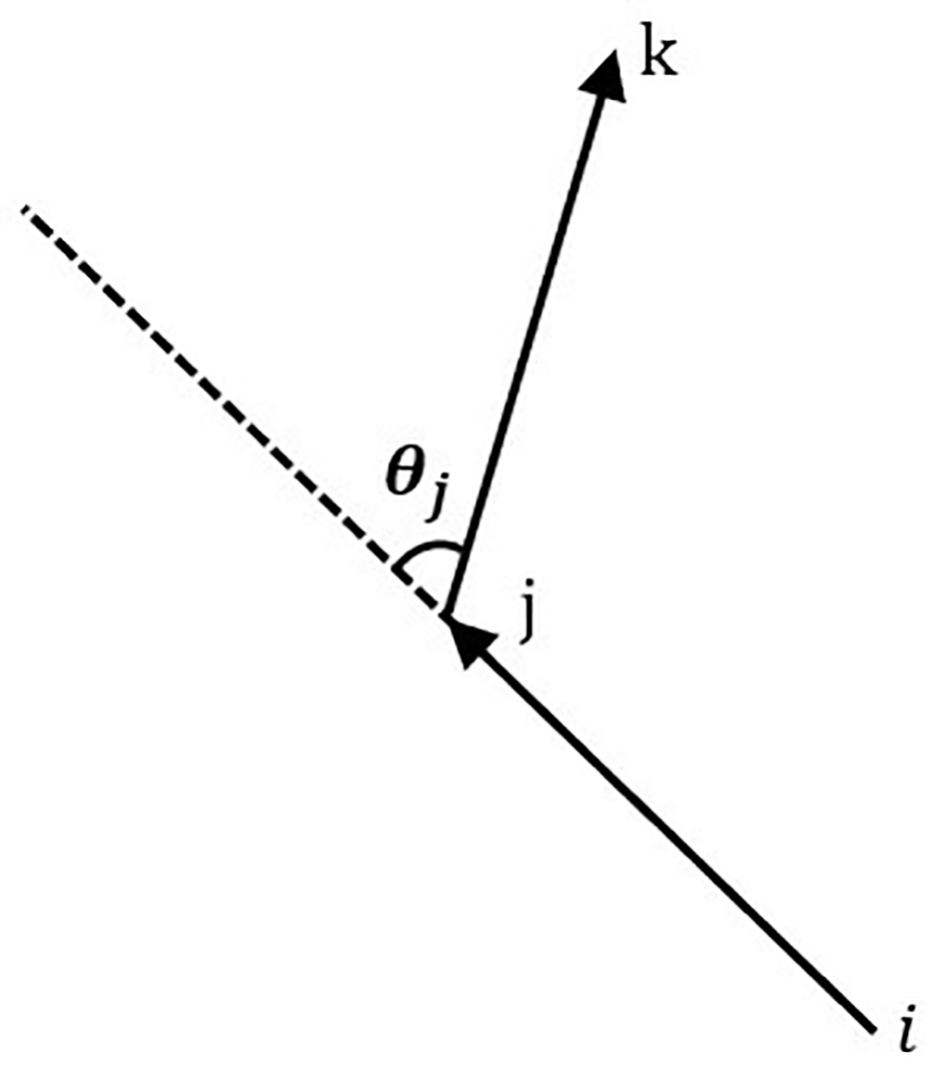
Diagram of the flight angle.

During the plant protection operation of the UAV, if the task nodes *i*, *j* and *k* are not in a straight line, there is a UAV steering phenomenon, that is, the UAV flies from node *i* to node *j*, and then flies to node *k* after turning. The angle between the extension line of line segment *ij* and line segment *jk* is called the steering angle of the UAV, denoted as *θ_j_*, as shown in **Figure 3**.

In the waypoint planning, the influence of the turning angle on the flight energy consumption of the UAV cannot be ignored (Fan et al., 2019). Therefore, the route planning considering the corner factor is particularly important for the plant protection operation of the UAV. The heuristic function in the traditional ACO algorithm only considers the influence of the distance between different nodes on the probability selection of the next node. In this study, the node corner factor is added to the heuristic information of the traditional ACO algorithm, so that the ants can choose a path with the smallest turning angle while ensuring the optimal path. The calculation equation of the corner evaluation function is as follows.


(5) $$R_{ijk}=c*\theta_{j}$$

where, *R_ijk_* is the turning angle factor;*c* is the turning angle weight coefficient; *θ_j_* is the turning angle.

The improved ACO can ensure that the ants take into account the dual factors of path length and turning angle when selecting nodes. The improved heuristic function equation is as follows.


(6) $$\eta_{ij}^{*}\left(t\right)=\frac{1}{d_{ij}+R_{ijk}}$$

#### 2.3.3 Pheromone update improvement

Ant colony algorithm is a parallel positive feedback computing mechanism, that is, each ant performs tasks independently according to the same mechanism. In ant colonies, some ants may find poor paths due to probabilistic problems, resulting in slow algorithm convergence, falling into local optimal solutions, and large fluctuations in results. Therefore, in order to improve the convergence speed and reduce the impact of inferior paths on the algorithm, a sorting optimization mechanism was added to the algorithm to update pheromone, that is, to enhance the pheromone concentration in the high-quality paths, thereby reducing the impact of inferior paths on the algorithm results.

Specifically, the sorting optimization mechanism will sort the paths traveled by all ants according to the path length (*L*_1_ ≤ *L*_2_ ≤···≤*L_m_*) after each iteration. The higher the ranking, the better the searched path. The pheromone weighting is carried out according to the ant’s ranking *μ*, and the pheromone on the high-quality path is enhanced, so that the ants are more inclined to choose the high-quality path with more pheromone in the next iteration. At the same time, according to the mechanism of elite ants, set the ant ranking *w*, only the first *w*−1 ants are allowed to release pheromone, the improved pheromone update equation are as follows.


(7) $$\tau_{ij}\left(t+1\right)=\rho \tau_{ij}\left(t\right)+\Delta \tau_{ij}^{*}\left(t,t+1\right)$$

(8) $$\Delta \tau_{ij}^{*}\left(t,t+1\right)=\overset{w-1}{\underset{k=1}{\sum }}\left(w-k\right)\Delta \tau_{ij}^{k}\;\left(t,t+1\right)$$

(9) Δ(t){QLk  Path (i,j) traversed by ant k  0     otherwise           

#### 2.3.4 Node cleaning

The planting methods of fruit trees are mostly arranged in a nearly straight line, that is, the arrangement of the fruit tree hearts in each row is close to a straight line. When UAV carry out plant protection operations, such arrangement of fruit trees can be regarded as a linear arrangement, and precise operations can be carried out by adjusting the spray width and spray direction of plant protection drone spray nozzles. However, in the path planning of such fruit trees, ACO has a large number of redundant nodes and unnecessary turning nodes that approximate straight line segments. Therefore, this study cleans the redundant nodes in the ACO algorithm by setting the error threshold.

**Figure 4:**
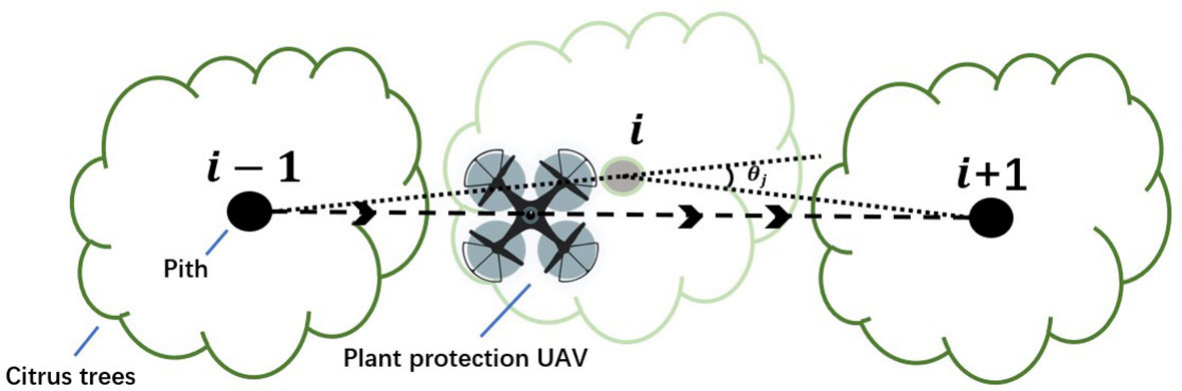
Redundant node clearing logic.

The step of node cleaning is set after planning the optimal path. According to the node coordinate information, the algorithm can obtain the rotation angle *θ_j_* of all nodes except the starting node and the end node. According to the node coordinate information, the algorithm can obtain the rotation angle *θ_j_* of all nodes except the starting node and the end node, and carry out the redundant node cleaning work through the set heading angle error threshold *φ*. For example, when cleaning and judging node *j*, the steering angle *θ_j_* of node *j* can be obtained from the angle between node *j* and the line connecting two adjacent nodes. When *θ_j_* ≤*φ*, it is considered that *θ_j_* is an invalid steering angle and is deleted; otherwise, node is preserved. The cleaning node logic is shown in **Figure 4**.

#### 2.3.5 Algorithm implementation process

**Figure 5:**
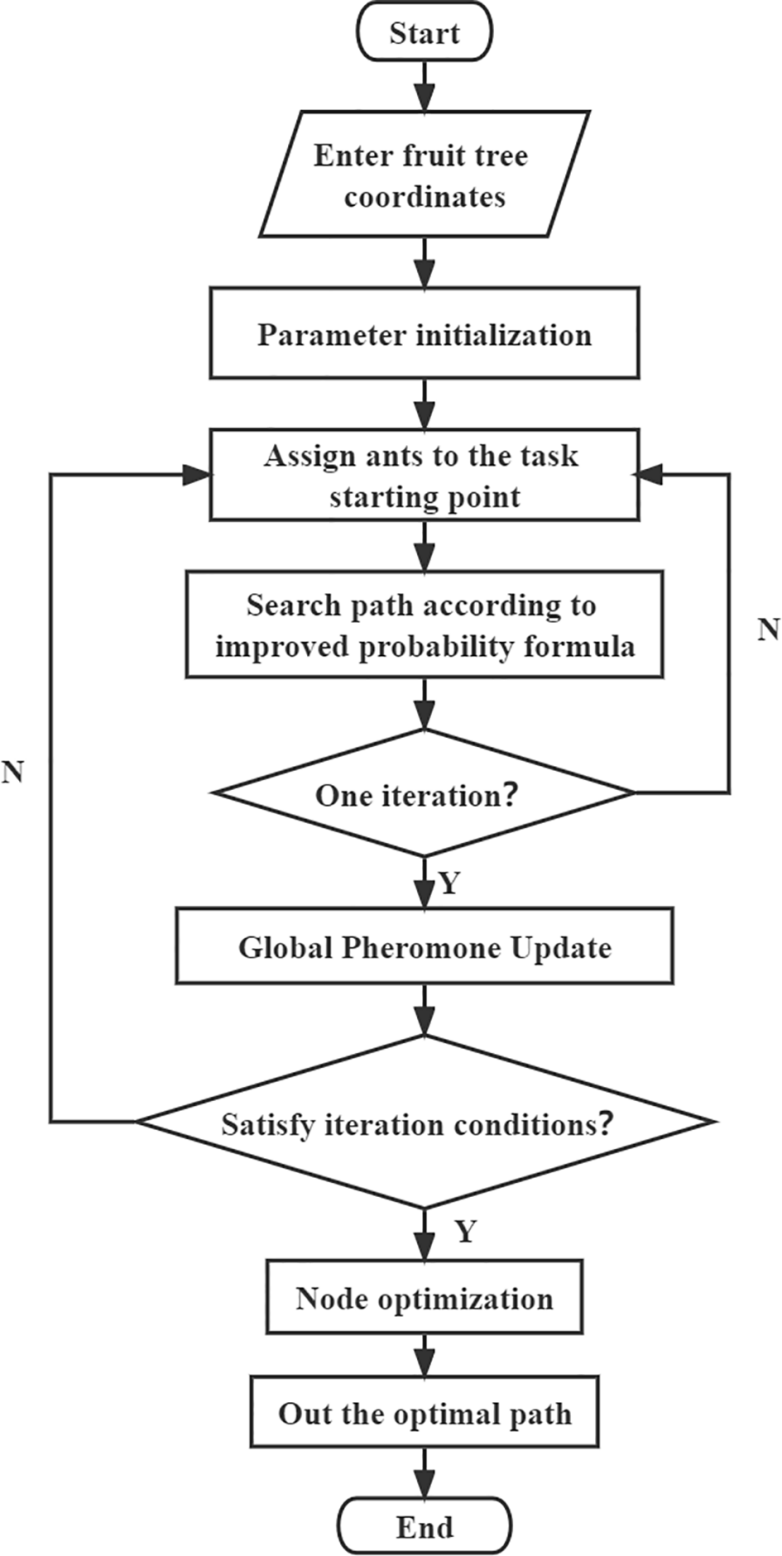
MS-ACO flow chart.

The path search process of MS-ACO is shown in **Figure 5**, and the pseudo code is shown in **Algorithm 1**. The algorithm mainly includes three important functions of task node search, pheromone update and node optimization, as follows:

(1). Task node search. Ants search for task nodes based on the improved heuristic function, and select task nodes through the dual factors of path length and corner factor.
(2). Pheromone update. After each iteration, the paths searched by all ants are sorted. Strengthen the pheromone concentration on the top-ranked paths, thereby improving the algorithm optimization ability.
(3). Node optimization. According to the optimization formula, node optimization is performed on the searched optimal path.

Algorithm 1. Pseudo code of MS-ACO 
Algorithm : MS-ACO.
1 **Output:** R_best_
2 **Input:** coordinate of nodes
3 initialize parameters
4 **while** *iter ≤ iter_max* **do**
5 put all ants to the starting point of the task
6 **for** each ant *k* **do**
7 **for** each node *i* **do**
8 compute *η_ij_
*(t) according to the formula (5) and (6)
9 choose next node with probability according to the formula (1) and (6)
10 **end for**
11 add node *i* to *tabu_k_
*
12 **end for**
13 computer the length of the shortest path
14 update the pheromone value according to the formula (7)
15 **end while**
16 for j=1 to length(tabu) do
17 if *θ_j_
*; ≤ *α* **then**
18 delete *node_j_
*
19 **else**
20 add node j to *R_best_
*
21 **end if**
22 **end for**

### 2.4 Simulation experiment

Simulation experiments were carried out in the MATLAB R2019b simulation environment, and 20 simulation experiments were carried out on the three experimental fields using ACO and MS-ACO. The simulation results of each test field are solved by averaging several experiments. The simulation results are compared in terms of path length, total angle, total node, and simulation path result graph to verify the feasibility and effectiveness of MS-ACO algorithm. There is no detailed theoretical method for the value of the parameters related to the ant colony algorithm, so this study repeated experiments to find the best values based on experience. The parameters of the ant colony algorithm mainly include the number of ants m, the rank of ants w, and the pheromone volatilization factor *ρ*, Pheromone importance factor *α*, Heuristic function importance factor *β*, Angle weight coefficient *c*, heading angle error threshold *φ*. The parameter values are shown in the **Table 1**.

**Table 1:** Related parameter values of improved ant colony algorithm.

Parameter	m	w	*ρ*	*α*	*β*	*c*	*φ*
Value	200~300	50	0.2	1	5	75~100	10

### 2.5 Field experiment

In order to further verify the real validity of the simulation experiment, we used the built UAV to conduct field experiments. The experiment time is September 17, 2022, the wind direction is north wind, the wind speed is 0.1m/s, the flying height of the drone is 7m, and the flight speed at the waypoint is 3m/s. The field experiments were analyzed from two aspects of flight trajectory and flight data. In the flight trajectory analysis, we used ArcMap software to project the GPS information recorded during the operation of the UAV to the orthophoto image of the orchard to analyze the trajectory of the UAV when it performed the route mission. ArcMap developed by Environment System Research Institute, has the functions of spatial analysis, map making, etc. It can match and transform the spatial coordinate system and is widely used in surveying and mapping (Wu et al., 2006). Digital orthophoto map is a kind of map containing geographic information, which has the characteristics of high precision, rich information and strong intuition (Fang, 2007). The flight data obtains the current, voltage and GPS information of the UAV during operation through the UAV flight log, and calculates the voyage time, range, turning angle and energy consumption per meter of the UAV during operation. The range and rotation angle are solved by basic geometric formulas. The calculation formula of the UAV’s flight energy consumption is as follows:


(10) P=UI

(11) $$W=\sum_{k=1}^{n}P_{k}\Delta t$$

where *P* is the instantaneous power of the drone, w. *U* is the working voltage of the drone, *V*. I is the sampling current of the drone, *A*. Δ*t* is the sampling time, *s*. *P_k_* is the power value collected at the *k*th moment, *w*. *W* is the flight energy consumption, *J*.

**Figure 6:**
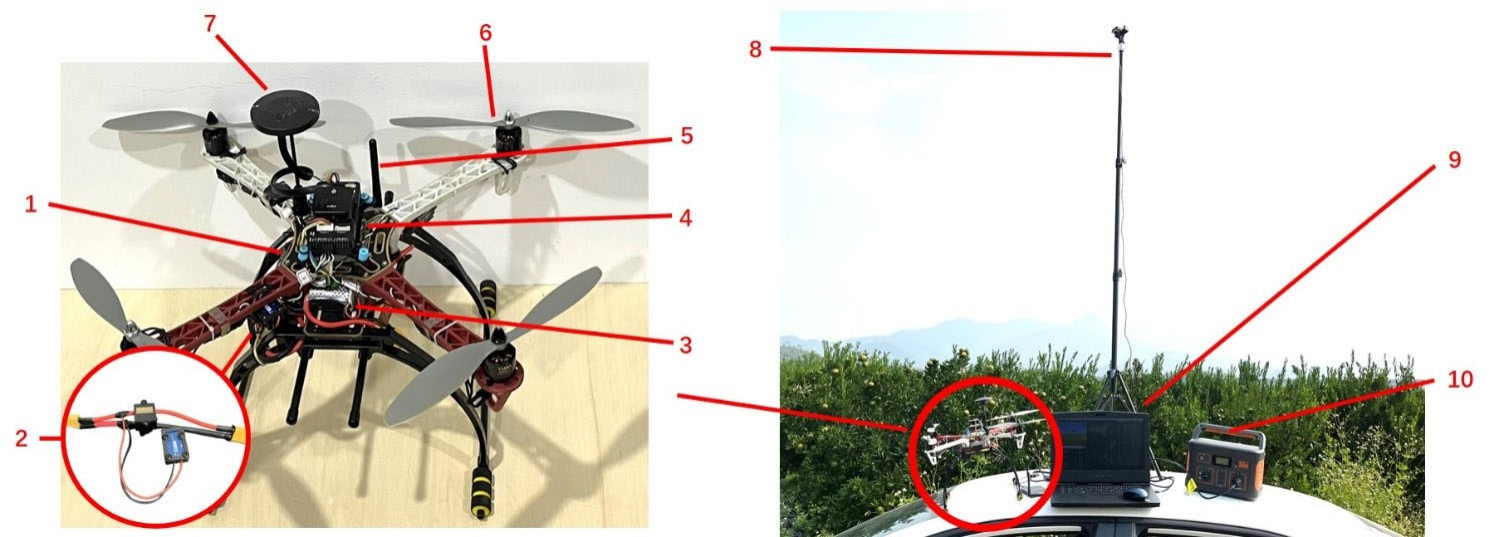
The real UAV (left) and UAV platform equipment (right). 1. Control signal receiver;2. MAUCH002+015 power module; 3. Power supply; 4. HEX Cube Black; 5. Data transmission module; 6. Brushless motor; 7. GPS module; 8. RTK base station; 9. Ground station; 10. Power bank.

A quadrotor small UAV was applied as a verification platform for waypoint path planning. The built small quadrotor UAV (left in **Figure 6**) includes: YH-2216 motor, HOBBYWING XRotor-20A -V1 -Asia Edition electronic governor, HEX MAUCH power module, 1047 carbon fiber propeller, F450 frame, HEX Cube Black flight control, Here+ positioning module. In the field test, in order to ensure the accuracy of UAV waypoint flight positioning and power detection, we use RTK base station for real-time dynamic differential positioning, and the positioning accuracy can reach 0.025m. The current and voltage module is the MAUCH002+015 power module, which uses the ACS758-200 version of the Hall current sensor, which can measure 200A of current with a measurement accuracy of ±3% (Zhang et al., 2021a). The four-rotor UAV cooperates with ground equipment to form a UAV flight platform. The built UAV flight platform is shown in **Figure 6** (right).

## 3 Result

### 3.1 Analysis of simulation results

According to the analysis of the statistical results in **Table 2**, it can be seen that MS-ACO has improved in both the optimal path and the total turning angle, among which the optimization effect of the total turning angle is the most obvious. Compared with ACO, the optimization effect of MS-ACO in the three experimental fields increased by 21.94%, 45.06% and 55.94% respectively. The main reason is that ACO searches the path through the path cost, which will cause the ants to fall into the local optimal solution in the densely planted area of citrus trees when searching for the path, increasing the total value of the path angle. In the path length optimization, MS-ACO is improved by 3.89%, 4.6% and 2.86% respectively compared with ACO, and the improvement effect is weak, which also shows that ACO has great advantages in path length optimization, and the improvement space is small. In terms of node optimization, the node optimization effect of the three experimental fields is obvious, all reaching more than 60%. It can be seen from the data in **Table 3** that the optimized total nodes and the total rotation angle show a positive correlation phenomenon, but there is no accurate correlation. The main reason is that each experimental field is not planted regularly, and the arrangement of fruit trees will have different degrees of influence on the optimization results. In general, ACO has certain advantages in path length optimization, but this algorithm is not suitable for the path planning of plant protection UAV in orchards. Although MS-ACO has a weak optimization ability in terms of path length, it has a strong ability to optimize the total turning angle of the path. Therefore, the overall optimization performance of MS-ACO is stronger than that of ACO, which is suitable for plant protection UAV orchard waypoint planning.

**Table 2:** Simulation results of traditional ant colony algorithm and improved ant colony algorithm.

Experimental field	MS-ACO			ACO			Optimization effect		
	Average path length	Average total turning angle	Total node	Average path length	Average total turning angle	Total node	Path length	Total corner	Total node
1	283.03	2686.69	37	294.47	3441.80	95	3.89%	21.94%	61.05%
4	493.12	3243.98	40	516.90	5904.77	159	4.60%	45.06%	74.84%
6	321.68	1986.32	26	331.14	4508.30	106	2.86%	55.94%	75.47%

**Table 3:** Statistical results of flight energy consumption.

Experimental field	MS-ACO				ACO				Optimization effect			
	Flight time/s	Voyage/m	Corner/°	Energy consumption per meter/kJ	Flight time/s	Voyage/m	Corner/°	Energy consumption per meter/kJ	Flight time	Voyage	Corner	Energy consumption per meter
Field 1	70.5	276.82	3403.81	0.128	131.7	282.26	6913.18	0.183	46.47%	1.93%	50.76%	30.05%
Field 4	95.7	485.38	4110.48	0.117	217.5	485.93	10753.95	0.176	56.00%	0.11%	61.78%	33.52%
Field 6	59.8	318.71	2445.59	0.119	145.9	311.33	8461.19	0.184	59.01%	-2.37%	71.10%	35.33%

**Figure 7:**
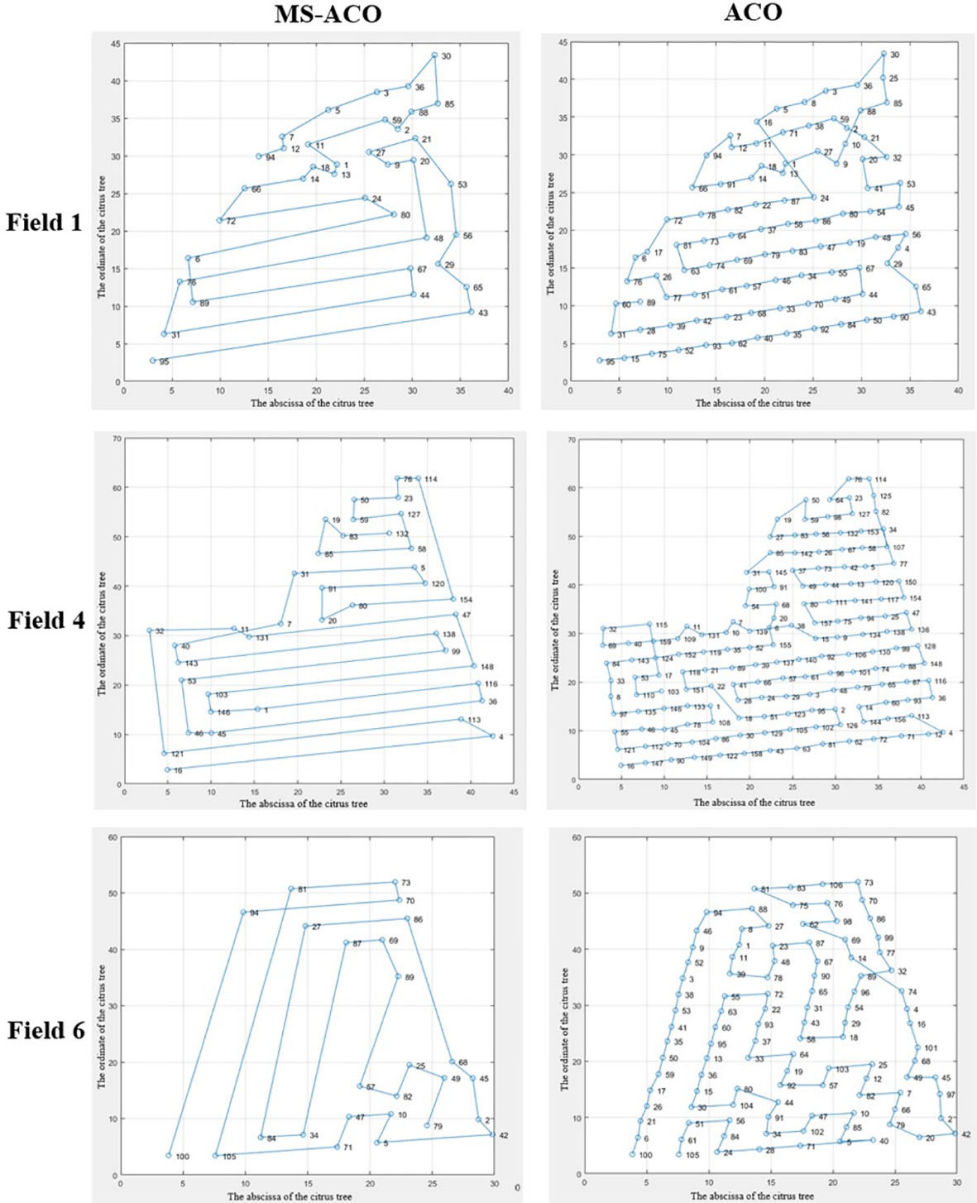
Comparison of the best paths for MS-ACO and ACO solutions.

**Figure 7** shows the optimal paths of MS-ACO and ACO in the three experimental fields. The first column in the figure is the optimal path for the MS-ACO to solve the three experimental fields, and the second column is the optimal path for the ACO to solve the three experimental fields. It can be seen from the pictures of the three experimental fields that the arrangement of citrus trees in each experimental field is roughly divided into regular areas and irregular areas. For example, the upper part of field 1 and field 4 are irregularly planted, the lower part is linearly arranged, the left side of field 6 is linearly arranged, and the right side is irregularly planted. The route optimized by MS-ACO roughly retains the advantages of full coverage reciprocating route planning in the regular planting area of citrus trees. However, unlike the full coverage reciprocating route method, MS-ACO can make accurate planning based on the coordinate information of fruit trees, and ensure that the route accurately covers the center of each fruit tree within the range of the tree center deviation threshold; in irregular areas, MS-ACO can more clearly highlight the advantages of its path optimization. Under the condition of ensuring the optimal route, the route is adjusted according to the position of each fruit tree. This also means that the UAV needs to make too many turns to accurately cover each fruit tree, which is inevitable. It can also be clearly seen in the ACO that the algorithm also roughly follows the planning characteristics of full coverage reciprocating routes. However, due to the intensive planting of citrus trees, ACO tends to fall into a local optimal solution, thus increasing the corner cost. At the same time, the increase of nodes also means an increase in the number of plant protection drones. The UAV will experience deceleration and acceleration when passing through each task node. Frequent acceleration and deceleration will greatly increase the energy consumption of the UAV and reduce the cruising time of the UAV. To sum up, although ACO has the advantage of the shortest path, the algorithm is not suitable for orchard waypoint planning due to too many task nodes and total turning angles. MS-ACO can not only better balance the cost of paths and corners, but also effectively reduce the number of task nodes, which has a strong advantage in orchard waypoint planning.

### 3.2 Experimental verification and discussion in the field environment

#### 3.2.1 Flight path analysis

**Figure 8:**
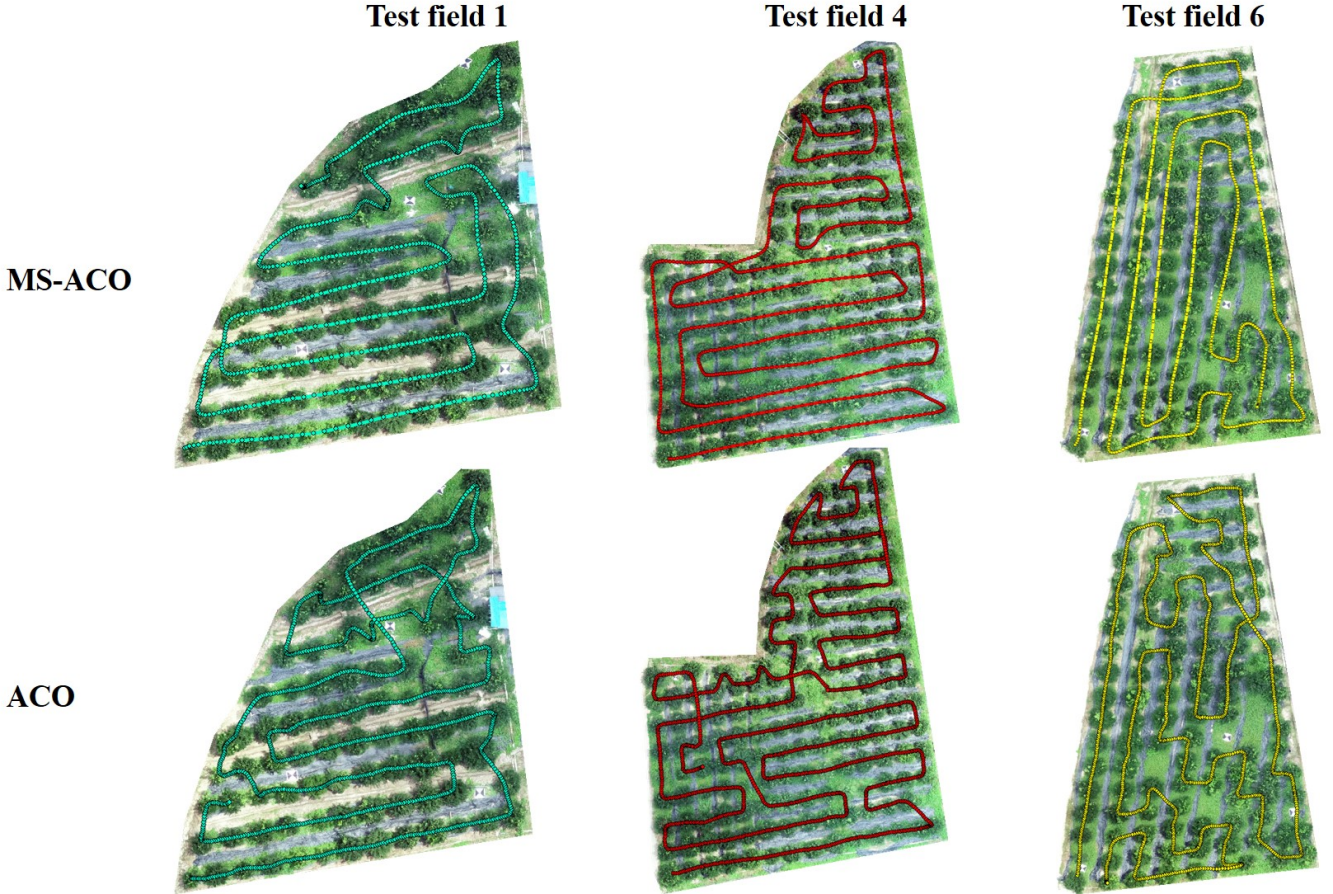
GPS flight trajectory of MS-ACO algorithm and ACO algorithm to solve the path.

**Figure 8** shows the GPS trajectory map of the UAV, which is composed of an orthophoto image and the UAV flight trajectory. In **Figure 8**, the first row is the flight trajectory of MS-ACO, and the second row is the flight trajectory of ACO. The blue, red and yellow colors represent the GPS trajectories of the three experimental fields respectively. Through the flight trajectory, we can better analyze the state of the UAV during operation. Here we mainly focus on the degree of coincidence between the trajectory and the fruit tree and the degree of fluctuation of the trajectory, which will directly affect the precision operation and flight energy consumption of the UAV. It can be seen from **Figure 8** that no matter the route optimized by MS-ACO or ACO, the flight trajectory and fruit trees have a high degree of overlap, indicating that the UAV can accurately execute the route task.For the degree of trajectory fluctuation, the route based on ACO optimization fluctuates greatly, which is not conducive to UAV plant protection operations. The route optimized based on MS-ACO is smoother and more in line with the flight characteristics of UAVs. The main factors affecting trajectory fluctuations are the mission nodes and turning angles in the route. When the UAV passes through each node, it will go through the process of acceleration and deceleration. Frequent acceleration and deceleration will lead to large fluctuations when the UAV is flying, thereby increasing unnecessary energy consumption, which is not conducive to UAV plant protection operations.

#### 3.2.2 Flight data verification

The statistical results of the flight data of the UAV are shown in **Table 3**. It can be seen that in the field experiment, the flight energy consumption of the path optimized by the MS-ACO is smaller than that of the ACO optimized path. For the three experimental fields, the voyage time optimization rates of MS-ACO are 46.47%, 56%, and 59.01%, respectively, and the energy consumption per meter optimization rates are 30.05%, 33.52%, and 35.33% respectively. Both optimization effects were significant and both improved significantly. The optimization effect of the voyage is less obvious, and even a negative value appears. The main reason is that the optimization effects of the two algorithms on the path are similar, and due to the influence of environmental factors (such as wind speed and GPS signal) in the field experiment, the flight stability of the UAV is poor, resulting in fluctuations in the data results. The optimization effect of the total turning angle of the path can reach more than 50%, but there are some differences. Because the planting of fruit trees is random, the optimization effect of the total turning angle will change with the distribution of fruit trees. The field experiment results show that compared with ACO, MS-ACO can greatly reduce the total turning angle of the UAV flight path, and the energy consumption per meter optimization rate can reach 30%. Although the voyage optimization effect is poor, it shortens the voyage time and reduces the flight energy consumption, which verifies the feasibility and effectiveness of MS-ACO in the waypoint planning in irregular orchards.

### 3.3 Comparison of simulation and field experiment results

**Figure 9:**
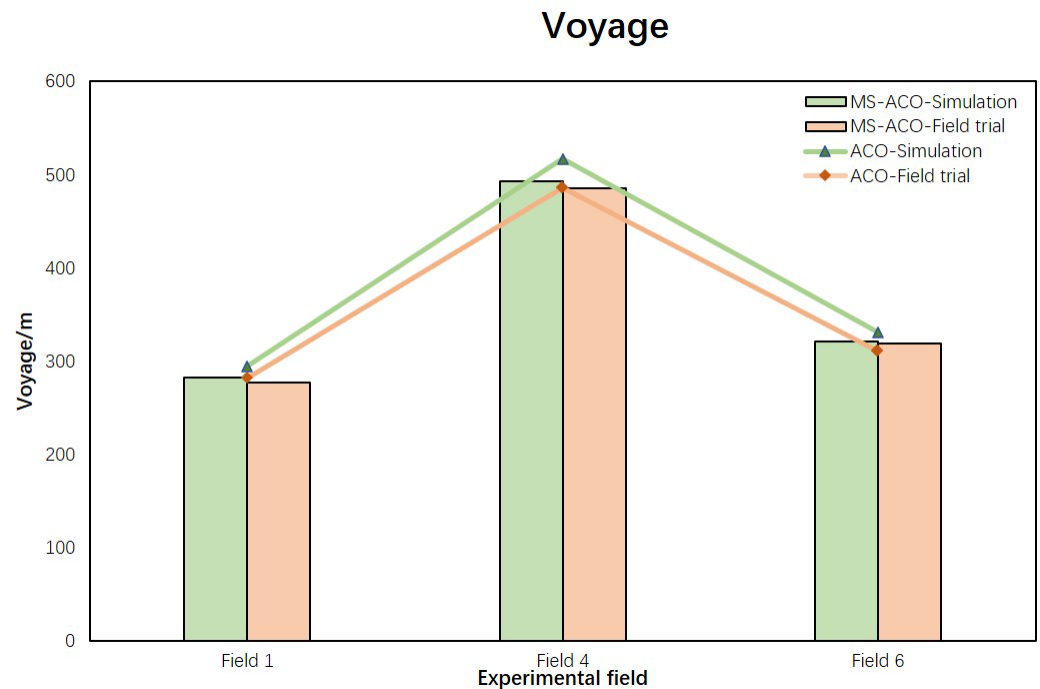
Comparison of path results between simulation experiment and field experiment.

**Figure 10:**
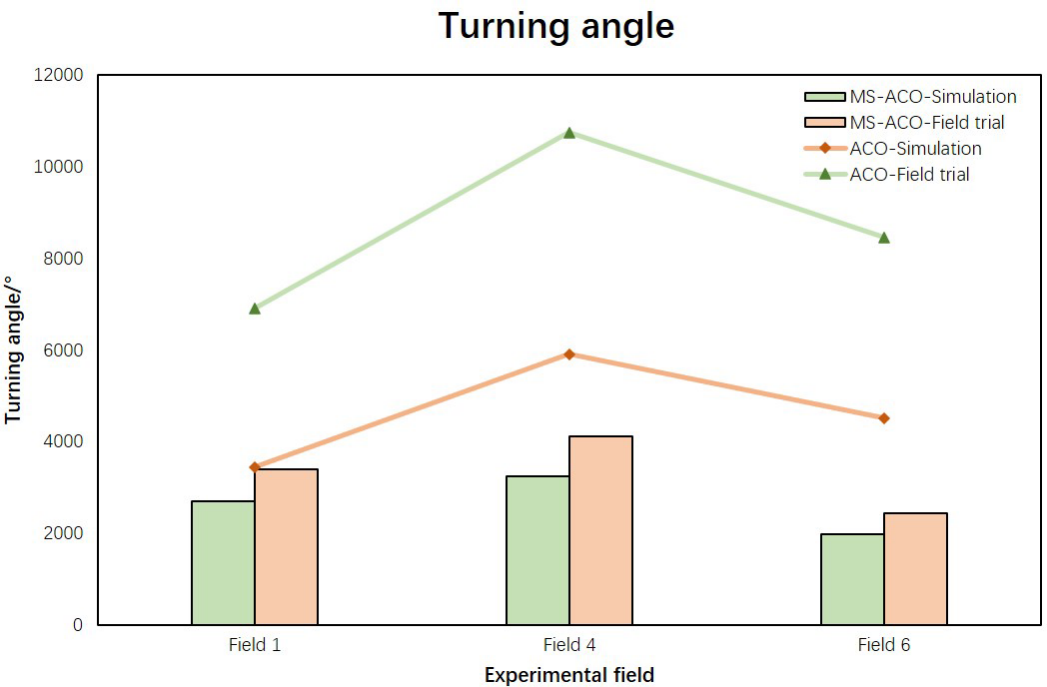
Comparison of corner results between simulation experiment and field experiment.

In the above two sections, we analyzed the optimization effects of the two algorithms in simulation experiments and field experiments respectively. In order to further verify the reliability of the improved algorithm, this section carries out visual analysis on the simulation results and experimental results of the algorithm from two parts: Voyage and Turning angle. The comparison of the results of the voyage is shown in **Figure 9**. The broken line part represents the result of the optimization of the ACO algorithm, and the bar part represents the result of the optimization of the MS-ACO algorithm. It can be seen that, whether it is a simulation experiment or a field experiment, the results of the two algorithms are not much different, indicating that the simulation results are consistent with the experimental results, and the UAV can perform the path flight mission well. For the corner optimization results (**Figure 10**), we can still see that the simulation experiment of the MS-ACO algorithm is not much different from the field experiment results, showing consistency. However, there is a big difference between the simulation experiment and the field experiment in the corner optimization effect of the ACO algorithm. The main reason is that there are too many path nodes planned by the ACO algorithm, and the UAV has frequent deceleration and acceleration when performing flight tasks, so the trajectory fluctuates greatly, which also causes a surge in the total path angle and increases the flight energy consumption. From this point, it can be shown that the path optimized by the ACO is not suitable for the waypoint planning of plant protection UAVs. In general, the results of MS-ACO both from simulation experiments and field experiments are within the error range, and the optimization results are relatively stable, which proves the reliability and effectiveness of the optimization path.

## 4 Discussion

Agricultural UAVs have received extensive attention in recent years, and related scholars have also carried out a lot of research on aerial plant protection technology, of which route planning is an important development direction for plant protection drones. At present, the flight path planning mainly focuses on the research on the coverage rate and operation efficiency of the farmland regulation degree, and lacks the waypoint planning for the fruit tree farmland. In this study, an exploration of waypoint path planning based on orchard was carried out to solve this problem. By improving the ant colony algorithm, the drone can achieve point-to-point precision flight operation of fruit trees.

At present, there are few researches on path planning for plant protection UAV node traversal. The ACO can quickly solve the shortest path, and it is easy to combine with other algorithms, which has a significant advantage in the waypoint planning of traversing nodes. Therefore, the ACO algorithm was used to carry out a preliminary exploration of the path optimization of the UAV waypoint. Most route planning studies use raster maps for environmental modeling. For example, Cheng et al. (2021) proposed an improved fusion algorithm of wolf swarm and particle swarm in order to solve the problems of premature convergence and local optimization of traditional particle swarm algorithm, which was effectively verified in the grid map constructed. Dai et al. (2019) combined the characteristics of MAX-MIN ant system and A* algorithm, proposed an improved ant colony algorithm to improve the high search ability of mobile robot complex map path, and verified it in grid map. Although the above research has been successful, the constructed grid map limits the freedom of movement of the robot, which has certain limitations in practical applications. In this study, a simulation environment is constructed according to the geographical coordinates of the orchard, and the optimal path is planned by combining the flight characteristics of the UAV and the position information of each fruit tree. It avoids the influence of grid maps, operation objects and other factors on the final path, and has practical application value.

This research focuses on improving the efficiency of plant protection UAV waypoint flight, including the flight time and energy consumption during UAV operation. In UAV plant protection operations, the primary issue affecting energy consumption is not the range, but the number of corners and waypoints in the path. In this study, the ACO algorithm was improved aiming at this problem. On the premise of ensuring the performance of ACO distance optimization, the corners, redundant nodes and iterations of the algorithm were optimized. The proposed MS-ACO can plan a reasonable spraying path based on the location information of multiple target fruit trees and integrate the three constraints of distance factor, turning angle factor and the number of task nodes. The results of simulation experiments and field experiments show that compared with the ACO algorithm, MS-ACO has been greatly improved in terms of corner optimization and node optimization, which enhances the operating efficiency of UAVs and reduces flight energy consumption. The distance difference between the two algorithms in the simulation experiment and the field experiment is within the error range, showing a high degree of consistency. However, there is a big difference in the total turning angle, and the total turning angle of the path planned by the ACO is multiplied. This further shows that although the path lengths planned by ACO and MS-ACO are not much different, the energy consumption and turning angle are greatly increased, and ACO does not have practical application value. However, the results of MS-ACO in the two sets of experiments are relatively stable, and the optimization of the corner and energy consumption is more consistent with the expected assumptions, and has good energy consumption optimization performance.

A preliminary investigation was conducted in this study on the waypoint planning of the plant protection UAV orchard scene, and the selected orchard is the orchard scene in the near-plain area. In such scenarios, elevation and slope of the orchard, and tree height have less impact on spraying efficiency. The proposed algorithm is capable of 2D optimal trajectory planning for such scenarios. In orchard areas with obvious height differences, elevation and tree height are one of the important factors affecting spraying efficiency. In the vast area of hilly mountainous terrain in China, orchard and tea gardens are commonly planted along the slopes (Wang et al., 2019; Liu et al., 2022). The path planning based on MS-ACO has more application value for such scenarios. Therefore, the waypoint planning for mountain and hilly scenes is one of the important directions of future research (Zheng et al., 2020). In the analysis of algorithm optimization, this study verified the quality of the results by comparing the optimization performance of ACO and MS-ACO algorithms. In future research work, we plan to carry out comparative research on other intelligent optimization algorithms to further improve the optimization performance of the MS-ACO algorithm and make it more generalizable. In addition, ACO has great advantages in solving combinatorial optimization problems. In the future research, it should also be combined with the dynamic changes of the load during the UAV spraying process, the battery life of the UAV, obstacles and other factors.

## 5 Conclusion

This study conducts research on the problem of UAV waypoint planning, and proposes an improved ant colony algorithm to solve the problem of point-to-point pesticide application in plant protection UAV orchard scenes. The improved algorithm incorporates the corner factor into the original heuristic function, which improves the corner optimization ability of the algorithm. At the same time, a ranking optimization mechanism is added to the pheromone update, which speeds up the convergence speed and avoids the influence of inferior solutions. Finally, the redundant nodes in the path are cleaned to further improve the energy consumption optimization rate of the algorithm. The method proposed in this study can carry out precision plant protection operation route planning according to the geographical location of each fruit tree. In the simulation and field experiment results, we have verified that the algorithm can plan a more low-consumption and efficient UAV plant protection route through performance indicators such as flight time optimization rate and energy consumption optimization rate per meter.

In future research, the influence of different obstacles in the field, take-off point and return point on the optimization rate of route energy consumption will be combined. At the same time, the environment perception of the sensor is used to improve the obstacle avoidance ability of the UAV during the route flight, making the algorithm more generalized and intelligent.

## Data availability statement

The original contributions presented in the study are included in the article/supplementary material. Further inquiries can be directed to the corresponding authors.

## Author contributions

YZ and YL: conceptualization, project administration, and funding acquisition. YZ, HT, and ZM: methodology. HT: software, data curation, and visualization. HT and YZ: validation and investigation. JX and HT: formal analysis. YZ: resources. CM, and YZ: writing—original draft preparation. YZ, YL, and RJ: writing—review and editing. YL: supervision. All authors contributed to the article and approved the submitted version.

## References

[B1] Abeywickrama H. V. Jayawickrama B. A. He Y. Dutkiewicz E. (2018). Comprehensive energy consumption model for unmanned aerial vehicles, based on empirical studies of battery performance. IEEE Access 6, 58383–58394. doi: 10.1109/ACCESS.2018.2875040

[B2] Bassanezi R. B. Lopes S. A. de Miranda M. P. Wulff N. A. Volpe H. X. L. Ayres A. J. (2020). Overview of citrus huanglongbing spread and management strategies in Brazil. Trop. Plant Pathol. 45 (3), 251–264. doi: 10.1007/s40858-020-00343-y

[B3] Cao R. Li S. Ji Y. Zhang Z. Xu H. Zhang M.. (2021). Task assignment of multiple agricultural machinery cooperation based on improved ant colony algorithm. Comput. Electron. Agr. 182, 105993. doi: 10.1016/j.compag.2021.105993

[B4] Cheng X. Li J. Zheng C. Zhang J. Zhao M. (2021). An improved PSO-GWO algorithm with chaos and adaptive inertial weight for robot path planning. Front. Neurorobot. 15, 770361. doi: 10.3389/fnbot.2021.770361 34803648 PMC8602895

[B5] Dai X. Long S. Zhang Z. Gong D. (2019). Mobile robot path planning based on ant colony algorithm with a* heuristic method. Front. Neurorobot. 13. doi: 10.3389/fnbot.2019.00015 PMC6477093 31057388

[B6] Du D. Lu L. Zhang L. Hu X. Huang Z. Chen G. (2011). Research progress in control technology of diaphorina citr. Chin. Agric. Sci. Bull. 27 (25), 178–181.

[B7] Ebadinezhad S. (2020). DEACO: Adopting dynamic evaporation strategy to enhance ACO algorithm for the traveling salesman problem. Eng. Appl. Artif. Intell. 92, 103649. doi: 10.1016/j.engappai.2020.103649

[B8] Erke S. Bin D. Yiming N. Qi Z. Liang X. Dawei Z. (2020). An improved a-star based path planning algorithm for autonomous land vehicles. Int. J. Adv. Robot. Syst. 17 (5), 1729881420962263. doi: 10.1177/1729881420962263

[B9] Fang J. (2007). Discussion on several technical problems about producting DOM. Geomatics Spatial Inf. Technol. 03), 91–93. doi: 10.3969/j.issn.1672-5867.2007.03.026

[B10] Fan Y. Wang D. Shen K. Zhang H. (2019). Design and experiment of the test system for steering energy consumption of electric multi-rotor unmanned aerial vehicle. Sci. Technol. Eng. 19 (10), 126–131. doi: 10.3969/j.issn.1671-1815.2019.10.019

[B11] Garcia A. G. Jamielniak J. A. Diniz A. J. F. Parra J. R. P. (2022). The importance of integrated pest management to flatten the huanglongbing (HLB) curve and limit its vector, the Asian citrus psyllid. Entomol. Gen. 42 (3), 349–359. doi: 10.1127/entomologia/2021/1247

[B12] Ghaderizadeh S. Abbasi-Moghadam D. Sharifi A. Tariq A. Qin S. (2022). Multiscale dual-branch residual spectral–spatial network with attention for hyperspectral image classification. IEEE J. Selected Topics Appl. Earth Observations Remote Sens. 15, 5455–5467. doi: 10.1109/JSTARS.2022.3188732

[B13] Han X. Kim H.-J. Jeon C.-W. Moon H. C. Kim J. H. Seo I.-H. (2021). Design and field testing of a polygonal paddy infield path planner for unmanned tillage operations. Comput. Electron. Agr. 191, 106567. doi: 10.1016/j.compag.2021.106567

[B14] Helmholz P. Rottensteiner F. Heipke C. (2014). Semi-automatic verification of cropland and grassland using very high resolution mono-temporal satellite images. ISPRS J. Photogramm. Remote Sens. 97, 204–218. doi: 10.1016/j.isprsjprs.2014.09.008

[B15] Huo F. Chi J. Huang Z. Ren L. Sun Q. Chen J. (2018). Review of path planning for mobile robots. J. Jilin University(Information Sci. Edition) 36 (06), 639–647. doi: 10.19292/j.cnki.jdxxp.2018.06.007

[B16] Hu P. Sharifi A. Tahir M. N. Tariq A. Zhang L. Mumtaz F.. (2021). Evaluation of vegetation indices and phenological metrics using time-series MODIS data for monitoring vegetation change in punjab, Pakistan. Water 13 (18), 2550. doi: 10.3390/w13182550

[B17] Jalayer S. Sharifi A. Abbasi-Moghadam D. Tariq A. Qin S. (2022). Modeling and predicting land use land cover spatiotemporal changes: A case study in chalus watershed, Iran. IEEE J. Selected Topics Appl. Earth Observ. Remote Sens. 15, 5496–5513. doi: 10.1109/JSTARS.2022.3189528

[B18] Lan Y. Chen S. Deng J. Zhou Z. Ou Y. (2019). Development situation and problem analysis of plant protection unmanned aerial vehicle in China. J. South China Agric. Univ. 40 (05), 217–225. doi: 10.7671/j.issn.1001-411X.201905082

[B19] Lan Y. Chen S. Fritz B. K. (2017). Current status and future trends of precision agricultural aviation technologies. J. Agric. Biol. Eng. 10 (3), 1–17. doi: 10.3965/j.ijabe.20171003.3088

[B20] Li J. Luo H. Zhu C. Li Y. Tang F. (2019). Research and implementation of combination algorithms about UAV spraying planning based on energy optimization. Trans. Chin. Soc. Agric. Machinery 50 (10), 106–115. doi: 10.6041/j.issn.1000-1298.2019.10.012

[B21] Lin J. Lan Y. Ou Y. Li J. Chen P. (2020). Construction and experimental verification of power consumption model for multi rotor agricultural UAV. J. Agric. Mechan. Res. 42 (05), 143–149. doi: 10.13427/j.cnki.njyi.2020.05.025

[B22] Lin N. Tang J. Li X. Zhao L. (2019). A novel improved bat algorithm in UAV path planning. CMC-Comput. Mater. Con. 61 (1), 323–344. doi: 10.32604/cmc.2019.05674

[B23] Liu C. Mao Q. Chu X. Xie S. (2019). An improved a-star algorithm considering water current, traffic separation and berthing for vessel path planning. Appl. Sci. Basel 9 (6), 1057. doi: 10.3390/app9061057

[B24] Liu Y. Ru Y. Liu B. Chen X. (2020). Algorithm for planning full coverage route for helicopter aerial spray. Trans. Chin. Soc. Agric. Eng. 36 (17), 73–80. doi: 10.11975/j.issn.1002-6819.2020.17.009

[B25] Liu Y. Zhang P. Ru Y. Wu D. Wang S. Yin N.. (2022). A scheduling route planning algorithm based on the dynamic genetic algorithm with ant colony binary iterative optimization for unmanned aerial vehicle spraying in multiple tea fields. Front. Plant Sci. 13. doi: 10.3389/fpls.2022.998962 PMC9523449 36186015

[B26] Niu W. Li Q. Li C. Cheng J. Ji D. Jiang X.. (2021). Multi-scale spatial variability and environmental drivers of soil nutrient distributions in the pearl river delta, south China. Ecol. Environ. Sci. 30 (04), 743–755. doi: 10.16258/j.cnki.1674-5906.2021.04.010

[B27] Patle B. K. Babu G. L. Pandey A. Parhi D. R. K. Jagadeesh A. (2019). A review: On path planning strategies for navigation of mobile robot. Defence Technol. 15 (4), 582–606. doi: 10.1016/j.dt.2019.04.011

[B28] Peng X. Lan Y. Hu J. Ou Y. Xiao K. Gao Z. (2019). Turning mode and whole region-coverage path planning and optimization of agricultural small UAV. J. South China Agric. Univ. 40 (02), 111–117. doi: 10.7671/j.issn.1001-411X.201805011

[B29] Ru Y. Liu Y. Qu R. Patel M. K. (2020). Experimental study on spraying performance of biological pesticides in aerial rotary cage nozzle. Int. J. OF Agric. AND Biol. Eng. 13 (6), 1–6. doi: 10.25165/j.ijabe.20201306.5511

[B30] Sharifi A. Mahdipour H. Moradi E. Tariq A. (2022). Agricultural field extraction with deep learning algorithm and satellite imagery. J. Indian Soc. Remote Sens. 50 (2), 417–423. doi: 10.1007/s12524-021-01475-7

[B31] Stasistics (2022). Available at: http://www.stats.gov.cn/tjsj/ (Accessed July 31, 2022).

[B32] Sun J. Tang J. Lao S. (2017). Collision avoidance for cooperative UAVs with optimized artificial potential field algorithm. IEEE Access 5, 18382–18390. doi: 10.1109/ACCESS.2017.2746752

[B33] Tang J. Liu G. Pan Q. (2021b). A review on representative swarm intelligence algorithms for solving optimization problems: Applications and trends. IEEE/CAA J. Automatica Sin. 8 (10), 1627–1643. doi: 10.1109/JAS.2021.1004129

[B34] Tang G. Tang C. Claramunt C. Hu X. Zhou P. (2021a). Geometric a-star algorithm: An improved a-star algorithm for AGV path planning in a port environment. IEEE Access 9, 59196–59210. doi: 10.1109/ACCESS.2021.3070054

[B35] Tariq A. Siddiqui S. Sharifi A. Shah S. H. I. A. (2022a). Impact of spatio-temporal land surface temperature on cropping pattern and land use and land cover changes using satellite imagery, hafizabad district, punjab, province of Pakistan. Arabian J. Geosciences 15 (11), 1045. doi: 10.1007/s12517-022-10238-8

[B36] Tariq A. Yan J. Gagnon A. S. Khan M. R. Mumtaz F. (2022b). Mapping of cropland, cropping patterns and crop types by combining optical remote sensing images with decision tree classifier and random forest. Geo. Spat. Inf. Sci. 1–19. doi: 10.1080/10095020.2022.2100287

[B37] Tian H. Fang X. Lan Y. Ma C. Huang H. Lu X.. (2022). Extraction of citrus trees from UAV remote sensing imagery using YOLOv5s and coordinate transformation. Remote Sens. 14 (17), 4208. doi: 10.3390/rs14174208

[B38] Wang D. Fan Y. Xue J. Yuan D. Shen K. Zhang H. (2019). Flight path control of UAV in mountain orchards based on fusion of GNSS and machine vision. Trans. Chin. Soc. Agric. Machinery 50 (04), 20–28. doi: 10.6041/j.issn.1000-1298.2019.04.002

[B39] Wu W. Li X. Zhang B. (2006). Probe of GIS developing technology based on ArcGIS engine. Sci. Technol. Eng. 6 (02), 176–178. doi: 10.3969/j.issn.1671-1815.2006.02.018

[B40] Wu S. Wen W. Wang C. Du J. Guo X. (2021). Research progress of digital fruit trees and its technology system. Trans. Chin. Soc. Agric. Eng. 37 (09), 350–360. doi: 10.11975/j.issn.1002-6819.2021.09.039

[B41] Xu L. Yang Z. Huang Z. Ding W. (2020). Route planning method for plant protection unmanned aerial vehicles combined with hy-brid particle swarm optimization. J. Chin. Comput. Syst. 41 (09), 1826–1832. doi: 10.3969/j.issn.1000-1220.2020.09.006

[B42] Yang S. Yang X. Mo J. (2018). The application of unmanned aircraft systems to plant protection in China. Precis. Agric. 19 (2), 278–292. doi: 10.1007/s11119-017-9516-7

[B43] Yi X. (2007). Discussion on the present situation and development trends of plant protection equipment in China. J. Agric. Mechanization Res. 03), 218–220. doi: 10.3969/j.issn.1003-188X.2007.03.071

[B44] Yu Y. Guan H. Li D. Jin S. Chen T. Wang C.. (2020). 3-d feature matching for point cloud object extraction. IEEE Geosci. Remote Sens. Lett. 17 (2), 322–326. doi: 10.1109/LGRS.2019.2918073

[B45] Zamani A. Sharifi A. Felegari S. Tariq A. Zhao N. (2022). Agro climatic zoning of saffron culture in miyaneh city by using WLC method and remote sensing data. Agriculture 12 (1), 118. doi: 10.3390/agriculture12010118

[B46] Zhang M. Ji Y. Li S. Cao R. Xu H. Zhang Z. (2020). Research progress of agricultural machinery navigation technology. Trans. Chin. Soc. Agric. Machinery 51 (04), 1–18. doi: 10.6041/j.issn.1000-1298.2020.04.001

[B47] Zhang D. Lan Y. Chen L. Wang X. Liang D. (2014). Current status and future trends of agricultural aerial spraying technology in China. Trans. Chin. Soc. Agric. Machinery 45 (10), 53–59. doi: 10.6041/j.issn.1000-1298.2014.10.009

[B48] Zhang H. Lerner E. Cheng B. Zhao J. (2021a). Compliant bistable grippers enable passive perching for micro aerial vehicles. IEEE-ASME T Mech 26 (5), 2316–2326. doi: 10.1109/TMECH.2020.3037303

[B49] Zhang H. Su Z. Hernandez D. E. Su B. (2018). Energy optimal path planning for mobile Robots based on improved AD* algorithm. Trans. Chin. Soc. Agric. Machinery 49 (09), 19–26. doi: 10.6041/j.issn.1000-1298.2018.09.002

[B50] Zhang Y. Tian H. Huang X. Ma C. Wang L. Liu H.. (2021b). Research progress and prospects of agricultural aero-bionic technology in China. Appl. Sci. Basel 11 (21), 10435. doi: 10.3390/app112110435

[B51] Zhang S. Yu J. Mei Y. Sun H. Du Y. (2017). Unmanned aerial vehicle trajectory planning by an integrated algorithm in a complex obstacle environment. Proc. Inst. Mech. Eng. G. J. Aerosp Eng. 231 (11), 2048–2067. doi: 10.1177/0954410016662058

[B52] Zhao D. Duan J. Chen P. Su J. (2017). Optimal path planning for 3D map based on a * algorithm. Comput. Syst. Appl. 26 (07), 146–152. doi: 10.15888/j.cnki.csa.005859

[B53] Zhao W. Wang Y. Zhang Z. Wang H. (2021). Multicriteria ship route planning method based on improved particle swarm optimization-genetic algorithm. J. Mar. Sci. Eng. 9 (4), 357. doi: 10.3390/jmse9040357

[B54] Zheng Y. Jiang S. Chen B. Lyu H. Wan C. Kang F. (2020). Review on technology and equipment of mechanization in hilly orchard. Trans. Chin. Soc. Agric. Machinery 51 (11), 1–20. doi: 10.6041/j.issn.1000-1298.2020.11.001

[B55] Zhou J. He Y. (2021). Research progress on navigation path planning of agricultural machinery. Trans. Chin. Soc. Agric. Machinery 52 (09), 1–14. doi: 10.6041/j.issn.1000-1298.2021.09.001

[B56] Zhou Z. Ming R. Zang Y. He X. Luo X. Lan Y. (2017). Development status and countermeasures of agricultural aviation in China. Trans. Chin. Soc. Agric. Eng. 33 (20), 1–13. doi: 10.11975/j.issn.1002-6819.2017.20.001

[B57] Zhou Z. Zang Y. Luo X. Yubin L. Xue X. (2013). Technology innovation development strategy on agricultural aviation industry for plant protection in China. Trans. Chin. Soc. Agric. Eng. 29 (24), 1–10. doi: 10.3969/j.issn.1002-6819.2013.24.001

[B58] Zhu L. Xu Z. Wang Y. Cheng C. (2022). Correlation analysis of energy consumption of agricultural rotorcraft. CMC-Comput. Mater. Con. 71 (2), 3179–3192. doi: 10.32604/cmc.2022.023293

